# Comprehensive Treatments for Congenitally Missing Teeth and Generalized Diastema

**DOI:** 10.1155/2017/3254873

**Published:** 2017-07-10

**Authors:** Mehran Bahrami, Fariba Saleh Saber, Amirreza Hendi

**Affiliations:** ^1^Dental Research Center, Department of Prosthodontics, Tehran University of Medical Sciences, School of Dentistry, Tehran, Iran; ^2^Department of Prosthodontics, Tabriz University of Medical Sciences, School of Dentistry, Tabriz, Iran; ^3^Department of Prosthodontics, Tehran University of Medical Sciences, School of Dentistry, Tehran, Iran

## Abstract

Congenitally missing teeth and/or hypodontia is a prevalent dental anomaly. There are different treatment options available for these conditions such as space maintenance, restoring the space by resin-bonded-fixed-partial-dentures (RBFPDs), and dental implants. This study addresses the comprehensive treatments for congenitally missing tooth and diastema using interdisciplinary approaches. One patient was treated with small-diameter-implants and the other one was treated using an intraoral scanner to make digital impression and fabricating RBFPDs with CAD/CAM system. Both patients were completely satisfied.

## 1. Introduction

Hypodontia is used to describe the congenital absence of one or more primary or secondary teeth. Excluding the third molars, the mandibular second premolars are the most frequently missing teeth, comprising between 60% to 72% of the total number of missing teeth [1].

The congenital absence of teeth and generalized diastema can seriously affect a young person both physically and emotionally, especially when the missing teeth and diastema are located in the anterior region of the mouth [2]. Complications associated with missing permanent teeth included malocclusion, periodontal problems, lack of alveolar-bone-growth, and unfavorable appearance [3].

The treatment options available for these patients included maintenance of the primary teeth, space closure by orthodontic treatment, space maintenance, restoring by RBFPDs, tooth transplantation, or dental implants [2].

Regardless of whether the location is posterior or anterior, RBFPDs have been accepted as an alternative treatment to conventional fixed-partial-dentures (FPDs) when intact abutments are present and minimal intervention is desired [4]. The main advantage of RBFPDs in comparison to conventional FPDs preparations is that they are conservative to tooth structure. So RBFPDs can be considered as the intermediate or definitive restorations following orthodontic treatment of congenitally absent teeth. The most common failure of RBFPDs is debonding and the patients should be aware of that [2].

Today, the first choice of restoration for a congenitally missing tooth should be a single-tooth implant [5]. The average implant platform, which is 4.0 mm wide, requires a minimum mesiodistal space of 1.0 mm between the platform and the adjacent tooth to facilitate proper healing and the development of interdental papilla. So a minimum of 6 mm space is required for the crown. The use of standard-diameter implants is common in implant treatments, but the lack of interdental space leads to unfavorable aesthetic results. Recently, the use of small-diameter implants has become more common [6]. Small-diameter implants (SDIs) or mini-dental implants (MDIs) generally are considered to be less than 3 mm in diameter. SDIs are introduced to overcome bone-quantity problems with a degree of success comparable to that of standard-diameter implants. The various designs of SDIs have become more commonly used in recent decades due to limitations in the geometry and capacity of the alveolar bone [6, 7].

Different impression materials and techniques have been used to achieve highly accurate conventional impressions [8]. Fabrication of definitive restorations through conventional methods involves a complicated process [9]. A relatively new approach employs Computer-Aided Design/Computer-Aided Manufacturing (CAD/CAM) technology to make a digital impression intraorally and design and produce the definitive restorations [10]. An increasing number of fixed prostheses are now produced by using intraoral digital impressions, which have become an important part of the digitization of prosthodontics [11].

The aim of this report is to present different treatment options available for congenitally absent teeth and discuss their advantages and disadvantages.

## 2. Case Report #1

**Figure 1:**
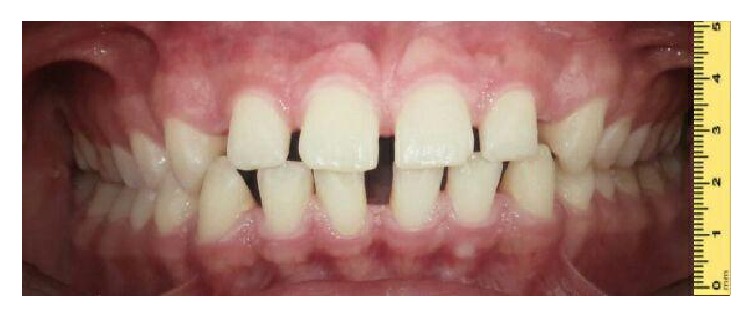
Intraoral frontal view before orthodontic treatment shows generalized diastema.

**Figure 2:**
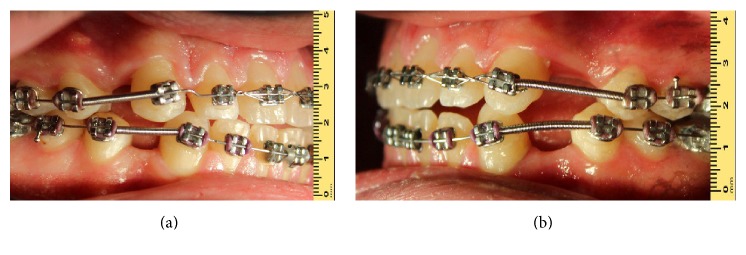
Intraoral view with fixed orthodontic appliance showing missing teeth and diastema. (a) Right side. (b) Left side.

A 23-year-old male patient with generalized diastema between the anterior teeth was referred to the Department of Prosthodontics (Figure 1). Fixed orthodontic treatment had been accomplished for about 3 years. Unfortunately, in the final stage of fixed orthodontic treatment, unequal spaces were available in the maxillary and mandibular first premolars (Figures 2(a) and 2(b)).

**Figure 3:**
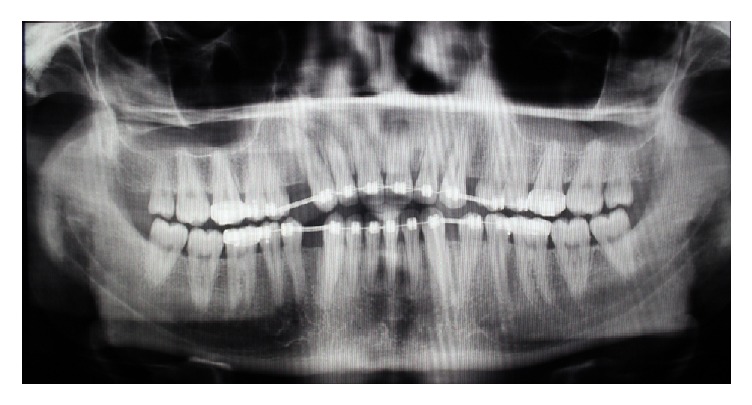
Panoramic view indicating root proximity between canines and second premolars in left side after orthodontic treatment.

**Figure 4:**
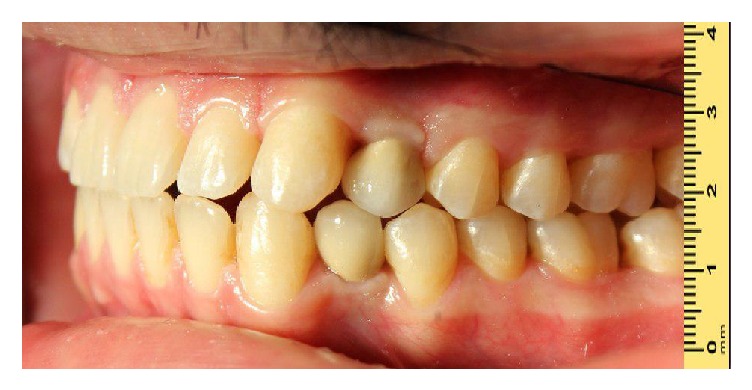
Final restorations for implants.

The patient did not have any systemic or genetic disorder associated with the diastema. The panoramic radiography revealed inadequate space in order to insert implant between the apices of canines and second premolars in maxillary and mandibular left sides (Figure 3). So, the option of implant was ruled out for the left side and the clinical decision was to fabricate RBFPDs. In the maxillary and mandibular right sides the mesiodistal spaces for the first premolar were adequate enough for placement of a single-tooth implant. According to the CBCT findings a 3.4*∗*12 mm fixture was inserted for tooth #5 and a 3.8*∗*12 mm fixture (Implantium, Internal hexagon connection, Dentium, Korea) was inserted for tooth #28. Three months later, the second stage surgery was performed and final restorations were fabricated. The metal-ceramic crowns were temporarily cemented with zinc-oxide-eugenol cement (Temp-Bond, Kerr, USA) (Figure 4).

**Figure 5:**
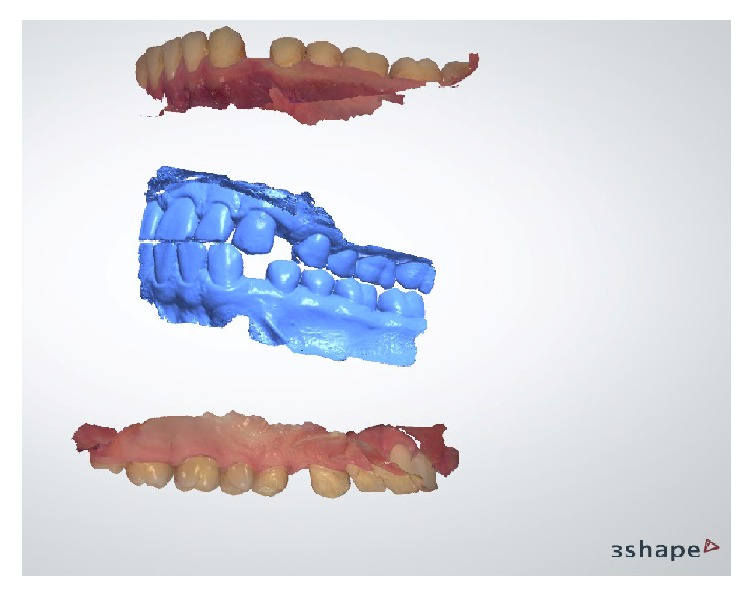
Intraoral digital impressions and interocclusal record.

**Figure 6:**
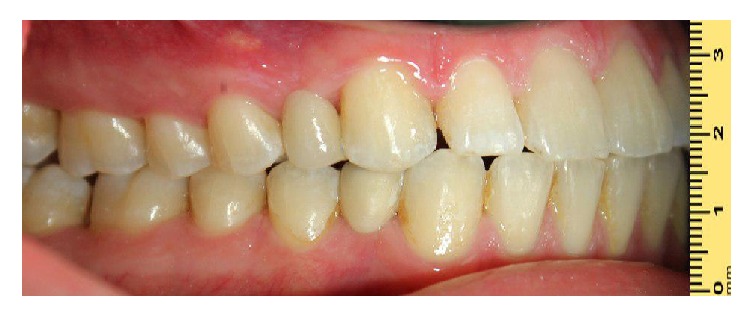
Resin-bonded-restorations delivery view.

For the mandibular RBFPDs, teeth #20 and 22 and, for maxillary RBFPDs, teeth #11 and 13 were prepared with chamfer finishing lines. The path of insertion was as paralleled as possible to optimize resistance and retention forms. In order to minimize metal-show-through, the incisal-finishing-line was prepared 2 mm short of the incisal edge, so that the incisal-edge-translucency was preserved from the facial aspect. A reduction of 0.5 mm lingually was prepared to allow the adequate bulk of metal to obtain enough strength of the retainers. A light chamfer-finishing-line was prepared 1 mm supragingivally. Supragingivally light chamfer maintains the preparation in the enamel which is essential for the optimal bonding. Subgingival margins may also cause some problems in digital systems. Interproximal-finishing-lines ended at the center of the contact areas. The preparation features correspond primarily to the optical-scanner-potentiality as well as to the milling machine capabilities. Undercut areas and small spikes or irregular surfaces on the preparation margin were eliminated. Digital impressions and inter-arch-records were made using 3shape intraoral scanner (3shape, Denmark) (Figure 5). RBFPDs frameworks were designed using 3shape dental system and the frameworks were sent to the laboratory. The frameworks were milled using Cr-Co blocks. The porcelain try-in was done and a mutually protected occlusion was selected for the patient. The RBFPDs were cemented using resin cement (Panavia, Kuraray America, Inc.) (Figure 6).

The patient was followed up every 6 months during a 2-year period.

## 3. Case Report #2

**Figure 7:**
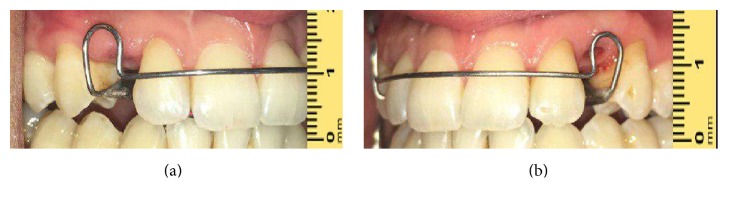
Intraoral view with removable orthodontic appliance showing missing teeth and diastema. (a) Right side. (b) Left side.

A 20-year-old female who had congenitally missing maxillary canines with anterior diastema in maxilla and crowding in anterior segment of mandible was referred to the Department of Prosthodontics. Removable-orthodontic-treatments were performed for maxillary and mandibular arches. One of the mandibular-central-incisors was extracted to gain sufficient space and to resolve the crowding. Fixed retainer was used to maintain the position of mandibular-anterior-teeth. In the maxilla, orthodontic removable retainer was used to eliminate the diastema by providing 3 mm spaces in the canine areas (Figures 7(a) and 7(b)). Clinical examination revealed inadequate space to insert regular implants between the laterals and first premolars in both sides. The minimal proximal preparation of the adjacent teeth was performed to increase the mesiodistal spaces to 4 mm. The RBFPD treatment was presented to the patient, but she refused to accept teeth preparation. As there was not enough mesial-distal space, one-piece SDIs, 2*∗*12 mm (SlimLine, Dentium, Korea), were considered to replace the maxillary canines.

Immediately following implant placement, provisional restorations were fabricated with temporary acrylic resin (UNIFAST™ LC, GC co). The provisional restorations had no centric or eccentric occlusal contact points.

**Figure 8:**
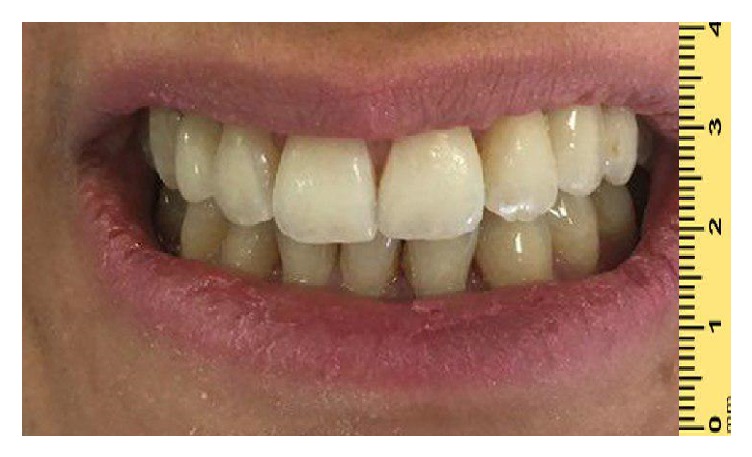
Final restoration delivery view.

The patient was followed up after one week. In this appointment, the sutures were removed. After a healing phase of three months, the final impression was taken with poly vinyl siloxane impression material (A-silicones, Kettenbach GmbH & Co. KG). The porcelain-fused-to-metal crowns were adjusted to have light occlusal contacts in centric occlusion. Partially group-function occlusion was given to the patient (Figure 8).

**Figure 9:**
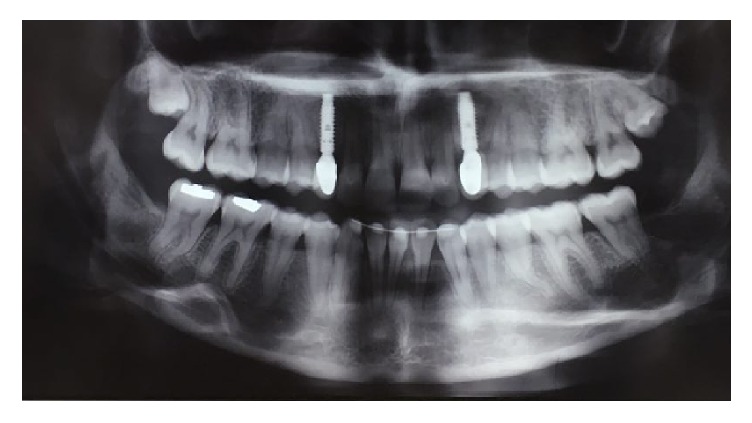
Panoramic image after two years of follow-up.

The patient was followed up every 6 months for two years (Figure 9).

## 4. Discussion

Comprehensive treatment of patients with missing teeth and/or hypodontia is difficult. It requires a teamwork including an orthodontist, a prosthodontist, and a surgeon (in case of implant insertions) to achieve ideal results.

Dental implants may be considered as the best treatment option for patients with hypodontia. However, using dental implants in patients with hypodontia may be challenging due to some limitations such as reduced mesiodistal space, poor bone quality and quantity (especially after orthodontic treatment), and compromised implants positions.

The orthodontist plays an important role in determining and establishing the space requirements for patients with missing teeth [12]. Then the prosthodontist should reassess the available space required for the implant fixture using the appropriate radiographs.

In some patients only SDIs should be considered. The survival rate of SDIs appears to be similar to that of regular diameter implants. Meta-analysis indicated an estimated 94.5% for 5-year survival of implant supported single crowns; therefore, the single-tooth implant has become a common treatment option for the replacement of congenitally absent teeth [6].

In patients with congenitally missing permanent teeth, orthodontic treatment is the gold standard [12]. However, orthodontic treatment can cause some potential risks and complications, because teeth undergone orthodontic movement may have resorption of cementum and dentine [13]. On the other hand, in some patients, it is impossible to insert even SDIs after orthodontic treatment because of the apices of the adjacent teeth roots. When adjacent teeth have been orthodontically moved in order to gain adequate space, radiographic examination often reveals either insufficient interradicular space available for an implant fixture or an absence of root parallelism [5]. In this condition there may not be adequate space even to place SDIs between the apices. So the use of RBFPDs will be recommended. Tooth reduction is conservative for RBFPDs preparation because of remaining in the enamel. This is one of the numerous advantages of this restoration; however, the three most common complications associated with RBFPDs are debonding (21%), tooth discoloration (18%), and caries (7%), although it has been reported that debonding does not appear to affect the patient's satisfaction and there is usually limited damage to abutment teeth [4, 14–16].

One major parameter for clinical success is the fit of a restoration [17]. The CAD/CAM systems are claimed to be more efficient than the conventional methods [18, 19]. Kugel et al. in an in vitro study showed that there were no significant differences in the marginal accuracy and fit of the crowns made by Lava COS and PVS impressions [20].

Recent systematic reviews have estimated that the five-year survival rates are 87.7% for RBFPDs and over 90% for conventional FPDs. Although these rates are lower than the 94.5% success reported for 5-year survival rate of implant supported single crowns, RBFPDs have the advantages of being less invasive and requiring a shorter total treatment time [14].

## 5. Conclusions

In the current case reports with congenitally missing teeth and diastema, optimal aesthetics and function were provided either by using small-diameter-implant-retained crowns or RBFPDs.

## Additional Points

*Key Messages*. Optimal aesthetics and function can be provided by using either small-diameter-implant-retained crowns or RBFPDs.
